# Synergistic antimycobacterial activity of *Piper longum* and *Cressa cretica* extracts and their effects on the expression of *rpoB*, *gyrA* and *gyrB* genes in *Mycobacterium tuberculosis*

**DOI:** 10.3892/mi.2026.345

**Published:** 2026-10-13

**Authors:** Vaibhav Kumar Tamrakar, Jyothi Bhat, Nitish Singh Parihar, Rajasubramaniam Shanmugam

**Affiliations:** 1 ICMR-National Institute of Research in Tribal Health (NIRTH), Jabalpur, Madhya Pradesh 482003, India; 2 Madhya Pradesh Medical Science University, Jabalpur, Madhya Pradesh 482003, India; 3 All India Institute of Medical Sciences Chhattisgarh, Raipur, Chhattisgarh 492099, India; 4 ICMR-National Institute of Traditional Medicine, Belagavi, Karnataka 590010, India; 5 Vels Institute of Science, Technology and Advanced Studies, Chennai, Tamil Nadu 600035, India

**Keywords:** *Mycobacterium tuberculosis*, *Piper longum*, *Cressa cretica*, synergistic activity, checkerboard assay, fractional inhibitory concentration index, gene expression, *rpoB*, *gyrA*, *gyrB*, medicinal plants

## Abstract

Tuberculosis (TB) remains one of the leading causes of mortality from infectious diseases worldwide, with the increasing prevalence of multidrug-resistant TB posing a major challenge to effective disease control. Combination therapy has become a key strategy for improving treatment efficacy, while minimizing the emergence of drug resistance. In the present study, the synergistic antimycobacterial activity of methanolic extracts of *Piper longum* (*P. longum*) and *Cressa cretica* (*C. cretica*) against *Mycobacterium tuberculosis* (*M. tuberculosis*; H37Rv) was investigated using a two-dimensional checkerboard assay. In addition, the effects of the individual extracts on the expression of the *rpoB*, *gyrA* and *gyrB* genes, which are associated with transcription and DNA replication, were evaluated using reverse transcription-quantitative PCR (RT-qPCR). The minimum inhibitory concentrations (MICs) of *P. longum* and *C. cretica* were 125 and 250 µg/ml, respectively. When used in combination, the MIC of *P. longum* decreased to 7 µg/ml, while that of *C. cretica* decreased to 10 µg/ml, corresponding to 18- and 25-fold reductions, respectively. The combination exhibited potent synergistic activity with a fractional inhibitory concentration index of 0.096. Phytochemical screening revealed the presence of several bioactive constituents, including alkaloids, phenolic compounds, flavonoids, tannins, terpenoids, glycosides, sterols, carbohydrates and proteins. RT-qPCR demonstrated that treatment with the plant extracts altered the expression profiles of *rpoB*, *gyrA* and *gyrB* during different stages of bacterial growth, with the most prominent transcriptional changes were observed between 24 and 72 h following treatment. On the whole, these findings demonstrate that *P. longum* and *C. cretica* exhibit significant synergistic antimycobacterial activity against *M. tuberculosis* and influence the expression of genes involved in essential cellular processes. Further bioactivity-guided fractionation, the identification of active phytochemicals, mechanistic investigations, cytotoxicity evaluation and *in vivo* studies are required to validate their potential as complementary therapeutic candidates for tuberculosis.

## Introduction

Tuberculosis (TB), caused by *Mycobacterium tuberculosis* (*M. tuberculosis*), remains one of the leading infectious diseases worldwide, despite decades of global control efforts. According to the World Health Organization Global Tuberculosis Report, ~10.8 million individuals developed TB and almost 1.25 million related deaths were reported worldwide in 2023, rendering TB the leading cause of mortality from a single infectious agent following the COVID-19 pandemic (1). The growing burden of multidrug-resistant (MDR) strains continues to threaten global TB elimination strategies (1-3). The emergence of drug resistance, prolonged treatment duration, adverse drug reactions and poor patient compliance has markedly reduced the effectiveness of currently available anti-TB treatments (2-5).

The standard treatment regimen for drug-susceptible TB consists of isoniazid, rifampicin, pyrazinamide and ethambutol (4). Highly effective, prolonged administration is frequently associated with hepatotoxicity, peripheral neuropathy, gastrointestinal disturbances, hypersensitivity reactions, nephrotoxicity and the emergence of drug-resistant strains (4,5). Resistance to first-line anti-TB drugs primarily results from chromosomal mutations in genes encoding drug targets; however, intrinsic resistance mechanisms, including reduced cell-wall permeability, efflux pump activity and antibiotic-inactivating enzymes, also contribute substantially to treatment failure (5,6). These challenges emphasize the urgent need to identify alternative therapeutic strategies capable of enhancing antimicrobial efficacy while minimizing toxicity and delaying the emergence of resistance.

Natural products have historically served as valuable sources of antimicrobial agents, and medicinal plants continue to provide structurally diverse bioactive compounds with significant therapeutic potential (7). Numerous phytochemicals, including alkaloids, flavonoids, phenolic compounds, terpenoids and glycosides, have demonstrated antimycobacterial activity against both drug-susceptible and drug-resistant *M. tuberculosis* (8-10). Moreover, several medicinal plants possess antioxidant and hepatoprotective properties that may help reduce drug-induced liver injury during prolonged anti-TB therapy (7,11). Combination therapy involving natural products has attracted considerable attention as synergistic interactions between phytochemicals may improve antimicrobial activity, lower effective drug concentrations, reduce toxicity and minimize the development of resistance (12,13).

*Piper longum* L. (*P. longum*; family Piperaceae), commonly known as long pepper or Pippali, is a major medicinal plant widely used in Ayurveda for the treatment of respiratory disorders (14). Its key bioactive constituent, piperine, possesses antimicrobial, anti-inflammatory, antioxidant, immunomodulatory and hepatoprotective properties (14,15). Previous research has also demonstrated that piperine enhances the activity of conventional antimycobacterial agents and may inhibit bacterial efflux pumps, thereby increasing intracellular accumulation of antimicrobial compounds (15). These characteristics render *P. longum* a promising candidate for the development of adjunctive therapies against TB.

Similarly, *Cressa cretica* L. (*C. cretica*; family Convolvulaceae), commonly known as Rudravanti, has long been used in traditional medicine for the treatment of respiratory and inflammatory disorders (16). The plant exhibits antibacterial, antioxidant, anti-inflammatory, analgesic, antipyretic and hepatoprotective activities, and has also been demonstrated to possess antimycobacterial potential (10,16). Although the individual antimycobacterial activities of *P. longum* and *C. cretica* have been reported, limited information is available regarding their combined effects against *M. tuberculosis* or their influence on molecular pathways associated with bacterial survival.

Among the essential genes required for the growth and survival of *M. tuberculosis*, *rpoB*, *gyrA* and *gyrB* encode critical proteins involved in transcription and DNA replication (17,18). The *rpoB* gene encodes the β-subunit of DNA-dependent RNA polymerase, the primary target of rifampicin, whereas *gyrA* and *gyrB* encode the two subunits of DNA gyrase, an essential type II topoisomerase responsible for DNA supercoiling, replication, transcription and chromosome segregation (17,18). Alterations in the expression or function of these genes are closely associated with bacterial adaptation and responses to antimicrobial stress (5,6).

The present study investigated the synergistic antimycobacterial activity of methanolic extracts of *P. longum* and *C. cretica* against *M. tuberculosis* H37Rv using a two-dimensional checkerboard assay. The present study further evaluated the effects of the individual plant extracts on the expression profiles of *rpoB*, *gyrA* and *gyrB* using reverse transcription-quantitative PCR (RT-qPCR) to obtain preliminary insight into the molecular responses of *M. tuberculosis* following exposure to these medicinal plant extracts.

## Materials and methods

### Plant collection and extraction

Taxonomically verified plant fresh leaves of *P. longum* (Voucher no. JN-BOT-1426) and *C. cretica* (Voucher no. JN-BOT-6532) were collected from the Herbal Garden of Jawaharlal Nehru Agricultural University, Jabalpur, Madhya Pradesh, India. The botanical nomenclature of both species was verified using the Tropicos database (https://www.tropicos.org) (19).

The collected leaves were washed thoroughly with distilled water, shade-dried at room temperature and pulverized into a fine powder using a mechanical grinder. Methanolic extracts were prepared using a Soxhlet extraction apparatus according to the method described in the study by Redfern *et al* (19). The extracts were concentrated under reduced pressure using a rotary evaporator and stored at 4˚C until further use.

### Qualitative phytochemical screening

Preliminary qualitative phytochemical analysis of the methanolic extracts of *P. longum* and *C. cretica* was carried out according to the standard phytochemical screening procedures described in the study by Ali *et al* (20). The extracts were screened for the presence of alkaloids, flavonoids, phenolic compounds, tannins, glycosides, carbohydrates, proteins, sterols, terpenoids, steroids and anthraquinones using established qualitative chemical tests (21).

### Bacterial strain and culture conditions

All microbiological experiments were performed in the Biosafety Level III (BSL-III) Tuberculosis Containment Laboratory of the Division of Communicable Diseases, ICMR-National Institute of Research in Tribal Health (ICMR-NIRTH), Jabalpur, India.

The reference strain *M. tuberculosis* H37Rv (ATCC 25618) was used throughout the study. The organism was cultured in Middlebrook 7H9 broth supplemented with 10% Oleic Acid-Albumin-Dextrose-Catalase (OADC; HiMedia Laboratories Private Limited) and 1% glycerol. Cultures were incubated at 37˚C under standard laboratory conditions until the required growth phase was achieved.

### Preparation of plant extract stock solutions

Stock solutions of the methanolic extracts were prepared in 1% methanol (cat. no. 320390, MilliporeSigma) containing 0.05% dimethyl sulfoxide (DMSO; cat. no. GRM5856, HiMedia Laboratories, LLC), as appropriate, to obtain a final concentration of 10 mg/ml (Table I). The solutions were sterilized by filtration through sterile 0.22-µm syringe filters (MilliporeSigma) and stored at 4˚C until use.

### Determination of minimum inhibitory concentration (MIC)

The antimycobacterial activity of the plant extracts was evaluated using the Resazurin Microtiter Assay (REMA), also referred to as the Colorimetric Redox Indicator Assay, following the standard protocol described in the study by Singh *et al* (22).

Briefly, 2-fold serial dilutions of each extract, ranging from 1.0 to 0.0625 mg/ml, were prepared in sterile 96-well microtiter plates. The bacterial inoculum was standardized to McFarland 1.0 and diluted 1:20 prior to inoculation. Each well received equal volumes of extract solution and bacterial suspension. Positive (bacterial growth) and negative (culture medium only) controls were included in every experiment.

Following incubation at 37˚C for 7 days, 30 µl of 0.02% resazurin solution were added to each well and the plates were incubated for an additional 24 h at 37˚C. A color change from blue to pink indicated bacterial growth, whereas the retention of the blue colour indicated the complete inhibition of growth. The MIC was defined as the lowest concentration of extract that prevented a change in color. All experiments were performed in triplicate and repeated independently.

### Evaluation of synergistic activity by checkerboard assay

The synergistic interaction between *P. longum* and *C. cretica* extracts was evaluated using a two-dimensional checkerboard microdilution assay, as previously described by Chen *et al* (12).

Serial two-fold dilutions of *P. longum* extract were dispensed along the horizontal axis of sterile 96-well microplates, whereas serial dilutions of *C. cretica* extract were arranged along the vertical axis, thereby generating various concentration combinations. Subsequently, 100 µl of a standardized bacterial suspension (1:20 dilution of McFarland 1.0 culture) were added to each well.

The final two columns of each plate served as positive (bacterial growth without extract) and negative (only media) controls. Wells containing individual extracts were included to determine their respective MIC values under identical experimental conditions.

Following incubation at 37˚C for 7 days, 30 µl of 0.02% resazurin solution (cat. no. P8833, MilliporeSigma) was added to each well, followed by an additional 24 h of incubation at 37˚C. Wells that remained blue were considered to exhibit complete inhibition of bacterial growth, whereas pink wells indicated bacterial proliferation.

### Fractional inhibitory concentration index (FICI)

The interaction between the two plant extracts was quantified by calculating the FICI according to Botelho (23). The FICI was calculated as follows:



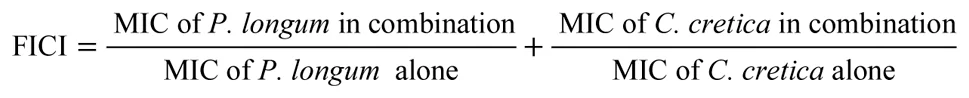



where the interaction was interpreted according to Odds (24) as follows: FICI ≤0.5, synergistic; 0.5 <FICI ≤4.0, additive or indifferent; and FICI >4.0, antagonistic.

### Gene expression analysis. RNA extraction and reverse transcription

For gene expression analysis, *M. tuberculosis* H37Rv cultures were grown in Middlebrook 7H9 broth (cat. no. 271310, BD DIFCO) supplemented with OADC. Individual plant extracts were added separately at sub-inhibitory concentrations corresponding to one-half of their respective MIC values. Untreated cultures served as the controls. Cultures were incubated at 37˚C and aliquots were collected at predefined time points (0, 6, 12, 24 and 72 h, as well as 7, 15 and 30 days) for RNA isolation.

Total RNA was extracted using TRIzol^®^ reagent (Invitrogen; Thermo Fisher Scientific, Inc.) according to the manufacturer's instructions and the method described in the study by Simões *et al* (25). Residual genomic DNA was removed by DNase I treatment and purified RNA was subsequently cleaned using RNeasy Mini spin columns (Qiagen, Inc.). RNA concentration and purity were determined spectrophotometrically. A total of 1.5 µg total RNA from each sample was reverse transcribed into complementary DNA (cDNA) using Sensiscript Reverse Transcriptase (cat. no. 205211, Qiagen, Inc.) according to the manufacturer's instructions. The reverse transcription conditions consisted of 70˚C for 5 min followed by 37˚C for 90 min. The synthesized cDNA was stored at -20˚C until qPCR analysis.

### qPCR

Gene expression analysis was performed using the QuantStudio™ 5 Real-Time PCR System (Applied Biosystems; Thermo Fisher Scientific, Inc.) with Luna Universal qPCR Master Mix (New England BioLabs, Inc.). A total of five primer pairs were used in the present study, including three target genes (*rpoB*, *gyrA* and *gyrB*) and two endogenous reference genes [16S rRNA and internal transcribed spacer (ITS); Table II]. Each 20-µL PCR reaction contained the following: 10 µl of 2X Luna Universal qPCR Master Mix (cat. no. M3003S, New England Biolabs), 1 µl each of forward and reverse primers, 1 µl of cDNA template and nuclease-free water to a final volume of 20 µl. Thermal cycling conditions consisted of an initial denaturation at 95˚C for 1 min, followed by 45 amplification cycles of 95˚C for 15 sec and 60˚C for 30 sec. A melt-curve analysis (65-95˚C) was performed following amplification to verify primer specificity and sample purity.

### Relative gene expression analysis

Relative gene expression was determined using SYBR-Green (cat. no. M3003S, New England Biolabs) chemistry. All reactions were performed in triplicate. The expression levels of the target genes were normalized against the endogenous reference genes (16S rRNA and ITS). Relative changes in gene expression between treated and untreated cultures were calculated using the comparative 2^-ΔΔCq^ method as previously described by Li *et al* (26,27).

### Statistical analysis

All experiments were performed independently in triplicate and statistical analyses were carried out using GraphPad Prism 7 (Dotmatics). For the gene expression data analysis, differences in relative gene expression were analyzed using two-way analysis of variance (ANOVA), with treatment and time as the two factors. Comparisons were made between the untreated control and each plant-extract-treated group at the corresponding time points. Post hoc comparisons were performed using Tukey's multiple-comparisons test. A P-value <0.05 was considered to indicate a statistically significant difference.

## Results

Qualitative phytochemical screening of *P. longum* and *C. cretica.* Preliminary qualitative phytochemical screening demonstrated that both methanolic extracts contained a diverse range of bioactive secondary metabolites (Table III). Alkaloids, phenolic compounds, tannins, flavonoids, glycosides, carbohydrates, proteins, sterols, anthraquinones and terpenoids were detected in both *P. longum* and *C. cretica*. The presence of these phytochemical classes suggests that the observed antimycobacterial activity may result from the combined action of multiple biologically active constituents rather than a single compound.

### MIC of the plant extracts

The antimycobacterial activity of the individual plant extracts was initially evaluated using the REMA. The MIC was defined as the lowest concentration that completely inhibited the color change of resazurin from blue to pink. The MIC values of the methanolic extracts were determined to be 0.125 mg/ml (125 µg/ml) for *P. longum* and 0.250 mg/ml (250 µg/ml) for *C. cretica* against *M. tuberculosis* H37Rv (Fig. 1). These concentrations were considered the effective inhibitory concentrations for subsequent synergistic interaction studies.

### Synergistic interaction between P. longum and C. cretica

To evaluate the interaction between the two plant extracts, a two-dimensional checkerboard assay was performed, followed by isobologram analysis (Fig. 2). A synergistic interaction was observed between the extracts (*P. longum* and *C. cretica*). In the presence of 0.125 mg/ml (125 µg/ml) *C. cretica*, the MIC of *P. longum* decreased from 0.125 mg/ml (125 µg/ml) to 0.007 mg/ml (7 µg/ml), representing an ~18-fold reduction. Likewise, the MIC of *C. cretica* decreased from 0.250 mg/ml (250 µg/ml) to 0.010 mg/ml (10 µg/ml) in the presence of 0.0625 mg/ml (62.5 µg/ml) *P. longum*, corresponding to an ~25-fold reduction.

The calculated FICI was 0.096, which is well below the accepted threshold for synergism (FICI ≤0.5). The isobologram demonstrated that all inhibitory concentration combinations were located below the line of additivity, confirming a strong synergistic interaction between the two extracts (Fig. 2). These findings indicate that the combination of *P. longum* and *C. cretica* enhanced the antimycobacterial activity of the individual extracts and substantially reduced the concentrations required to inhibit the growth of *M. tuberculosis*.

### Effect of plant extracts on the expression of rpoB, gyrA and gyrB

To investigate the transcriptional response of *M. tuberculosis* following exposure to the plant extracts, the relative expression of *rpoB*, *gyrA* and *gyrB* was analyzed using RT-qPCR at different time points following treatment with the individual extracts (Fig. 3). Treatment with both *P. longum* and *C. cretica* resulted in time-dependent changes in the expression profiles of all three target genes. Changes in relative transcript abundance were observed during the early stages of exposure, with pronounced changes occurring between 24 and 72 h.

The expression profiles differed according to the target gene, plant extract and duration of exposure. For *rpoB*, treatment with *P. longum* resulted in a pronounced change in relative expression, with the highest level observed at 48 h, followed by a gradual decrease at later time points. A similar temporal pattern was observed following treatment with *C. cretica*, although the magnitude of the change was lower than that observed with *P. longum* (Fig. 3A).

The expression of *gyrA* also exhibited a time-dependent pattern, with the highest relative expression observed at 72 h following treatment with both extracts. The expression subsequently decreased at 7 and 15 days and approached the control level at 30 days (Fig. 3B). For *gyrB*, the most notable change was observed at 48 h, followed by a progressive decrease during the subsequent time points. The response to *P. longum* was generally greater than that observed with *C. cretica* (Fig. 3C).

Two-way ANOVA demonstrated significant effects of treatment and time on the expression of *rpoB*, *gyrA* and *gyrB*, with a significant treatment vs. time interaction for all three genes. Post hoc comparisons using Tukey's multiple-comparisons test identified significant differences between the treatment groups at several time points, as shown in Fig. 3.

The temporal expression patterns indicate that exposure to the plant extracts was associated with changes in the transcription of genes involved in essential cellular processes, including transcription (*rpoB*) and DNA replication (*gyrA* and *gyrB*). The differences in the magnitude and timing of these responses suggest that the transcriptional response varied according to both the extract and the duration of exposure. These changes in transcript abundance represent a molecular response to phytochemical exposure.

## Discussion

TB continues to represent a major global public health challenge despite the availability of effective treatment (4). The emergence of MDR and extensively drug-resistant (XDR) *M. tuberculosis* strains, together with prolonged treatment regimens, poor patient compliance and drug-associated toxicity, has considerably reduced the effectiveness of current therapeutic strategies (1-4). Consequently, there is increasing interest in identifying novel antimycobacterial agents from natural sources and developing combination therapies capable of enhancing antimicrobial efficacy while reducing the risk of resistance development (26).

The present study demonstrated that the methanolic extracts of *P. longum* and *C. cretica* possess significant antimycobacterial activity against *M. tuberculosis* H37Rv when tested individually. More notably, the checkerboard assay revealed a pronounced synergistic interaction between the two extracts, resulting in substantial reductions in the MICs of both plant extracts. Specifically, the MIC of *P. longum* decreased from 125 to 7 µg/ml, whereas the MIC of *C. cretica* decreased from 250 to 10 µg/ml, yielding a FICI of 0.096. According to the interpretative criteria proposed by Odds (23), an FICI value ≤0.5 is indicative of synergism. The exceptionally low FICI obtained in the present study indicates a potent synergistic interaction and suggests that the combination of these extracts is considerably more effective than either extract alone.

The potential of plant-derived compounds to act synergistically has attracted considerable attention in antimicrobial research as phytochemicals frequently target multiple bacterial pathways simultaneously (26,27). Unlike conventional antibiotics, which generally interact with a single molecular target, crude plant extracts contain structurally diverse bioactive constituents capable of producing complementary or additive biological effects (27). Such multitarget activity is particularly advantageous against *M. tuberculosis*, whose lipid-rich cell envelope, intrinsic resistance mechanisms and metabolic adaptability often limit the efficacy of single-agent therapies (27). Therefore, the marked reduction in MIC observed in the present study supports the concept that combining medicinal plants may enhance antimycobacterial efficacy while reducing the concentrations required for bacterial inhibition.

Preliminary phytochemical analysis demonstrated that both extracts contained several classes of secondary metabolites, including alkaloids, flavonoids, phenolic compounds, tannins, terpenoids, glycosides, sterols, carbohydrates and proteins. Many of these phytochemical classes have previously been reported to exhibit antimycobacterial activity through diverse mechanisms, including the disruption of the mycobacterial cell envelope, interference with nucleic acid synthesis, the inhibition of essential enzymes, the induction of oxidative stress and the modulation of bacterial metabolic pathways (28). Since crude plant extracts comprise numerous bioactive constituents, the synergistic activity observed in the present study is likely attributable to the combined action of multiple phytochemicals rather than a single active compound.

Among the phytochemicals identified in *P. longum*, piperine is considered a principal bioactive alkaloid associated with several of the pharmacological properties of the plant. Piperine exhibits antimicrobial, antioxidant, anti-inflammatory, immunomodulatory and hepatoprotective activities (14,15). Piperine has been shown to inhibit bacterial efflux pumps, alter membrane permeability and enhance the intracellular accumulation of antimicrobial agents, thereby potentiating their antibacterial efficacy (15,29). These mechanisms were not directly investigated in the present study; the marked reduction in MIC observed during combination treatment suggests that similar complementary interactions between constituents of *P. longum* and *C. cretica* may contribute to the enhanced antimycobacterial activity.

Likewise, *C. cretica* contains diverse phenolic compounds, flavonoids and other secondary metabolites that possess antimicrobial, antioxidant, anti-inflammatory and hepatoprotective activities (16,28). These phytochemicals may complement the activity of piperine by targeting distinct bacterial pathways, thereby producing additive or synergistic biological effects. Such multitarget interactions are particularly advantageous for chronic bacterial infections as simultaneous interference with multiple physiological processes reduces the probability of resistance development (27,30).

The precise mechanism responsible for the observed synergistic interaction remains to be elucidated. The enhanced activity may result from increased membrane permeability, the inhibition of bacterial efflux systems, the disruption of cell wall integrity, interference with essential metabolic pathways or the simultaneous action of multiple phytochemicals on distinct molecular targets (30). Alternatively, one extract may facilitate the intracellular availability or activity of bioactive constituents present in the other extract. Nevertheless, these mechanisms remain speculative and require confirmation through bioactivity-guided fractionation, metabolomic profiling, enzyme inhibition studies and transcriptomic analyses.

To further investigate the molecular response of *M. tuberculosis* to phytochemical exposure, the expression of the essential genes *rpoB*, *gyrA* and *gyrB* was evaluated using RT-q PCR. These genes encode proteins that play indispensable roles in bacterial transcription and DNA replication and are well-established molecular targets of first-line and second-line anti-TB drugs, including rifampicin and fluoroquinolones (31). Consequently, changes in the expression of these genes may provide valuable insight into the bacterial response following exposure to antimicrobial agents.

The present study demonstrated that treatment with the methanolic extracts of *P. longum* and *C. cretica* resulted in time-dependent changes in the expression of *rpoB*, *gyrA* and *gyrB*. The most pronounced changes were observed between 24 and 72 h following treatment, followed by a gradual reduction in relative transcript abundance at later time points. The magnitude and timing of the response differed among the three genes and between the two extracts, with *P. longum* generally producing more notable changes in relative expression than *C. cretica*. These findings indicate that exposure to the plant extracts elicited measurable transcriptional responses in genes associated with essential cellular processes. The significant effects of treatment and time, together with the treatment vs. time interaction observed, further indicate that the transcriptional response was dependent on both the extract and the duration of exposure.

The changes in transcript abundance should not be interpreted as direct evidence of inhibition of RNA polymerase or DNA gyrase activity. Instead, they are more likely to reflect adaptive molecular responses to phytochemical-induced stress. Bacterial exposure to antimicrobial agents is known to trigger transcriptional reprogramming involving genes associated with DNA replication, transcription, stress adaptation and metabolic homeostasis as part of a coordinated physiological response to environmental stress (29,32). Therefore, the differential expression profiles observed in the present are consistent with a transcriptional response of *M. tuberculosis* to phytochemical exposure rather than providing direct evidence of enzyme inhibition. Further investigations integrating transcriptomics, proteomics and enzyme activity assays will be required to determine the functional significance of these transcriptional alterations.

The temporal changes in gene expression observed in the present study indicate an early molecular response to phytochemical exposure. The marked changes observed between 24 and 72 h, followed by reduced transcript abundance at later time points, suggest that the transcriptional response may vary during prolonged exposure. Bacterial exposure to antimicrobial agents can involve early transcriptional reprogramming followed by downstream physiological effects on cellular growth and survival (29,32). Thus, the gene expression profiles observed herein provide preliminary evidence of a time-dependent molecular response to the individual plant extracts. However, the association between these transcriptional changes and the observed antimycobacterial activity requires further investigation.

The present study has several limitations which should be mentioned. Gene expression analysis was performed using the individual methanolic extracts of *P. longum* and *C. cretica* rather than their synergistic combination, and phytochemical analysis was limited to qualitative screening without identification of the active constituents. Future studies are required to evaluate the transcriptional effects of the extract combination and employ bioactivity-guided fractionation with HPLC, LC-MS, GC-MS and NMR to identify the compounds responsible for the observed synergistic activity. The antimycobacterial activity was evaluated only against the laboratory strain *M. tuberculosis* H37Rv. Validation using clinical MDR/XDR isolates, together with cytotoxicity, hepatoprotective and *in vivo* studies, is required to establish the therapeutic efficacy and safety of this plant combination. Despite these limitations, the marked reduction in MIC values and the observed modulation of essential bacterial genes support the potential of the *P. longum-C. cretica* combination as a promising phytotherapeutic candidate for TB, warranting further mechanistic and preclinical investigations.

In conclusion, the present study demonstrates that the methanolic extracts of *P. longum* and *C. cretica* possess significant antimycobacterial activity against *M. tuberculosis* H37Rv and exhibit strong synergism in combination (FICI, 0.096), with marked reductions in the MICs of both extracts. Phytochemical screening identified alkaloids, flavonoids, phenolics, tannins, terpenoids and glycosides, which may collectively contribute to the observed synergistic effect. The RT-qPCR analysis revealed the time-dependent modulation of the essential *rpoB*, *gyrA* and *gyrB* genes following treatment with the individual extracts, with the most pronounced transcriptional changes observed during the early exposure period. These findings provide preliminary molecular evidence of a transcriptional response to phytochemical exposure. However, the observed changes in transcript abundance do not establish the direct inhibition of RNA polymerase or DNA gyrase activity. Further studies involving the combined extracts, clinical *M. tuberculosis* isolates, the identification of active phytochemicals, protein-level analysis and functional enzyme assays are required to elucidate the mechanisms underlying the observed synergistic antimycobacterial activity.

## Figures and Tables

**Figure 1:**
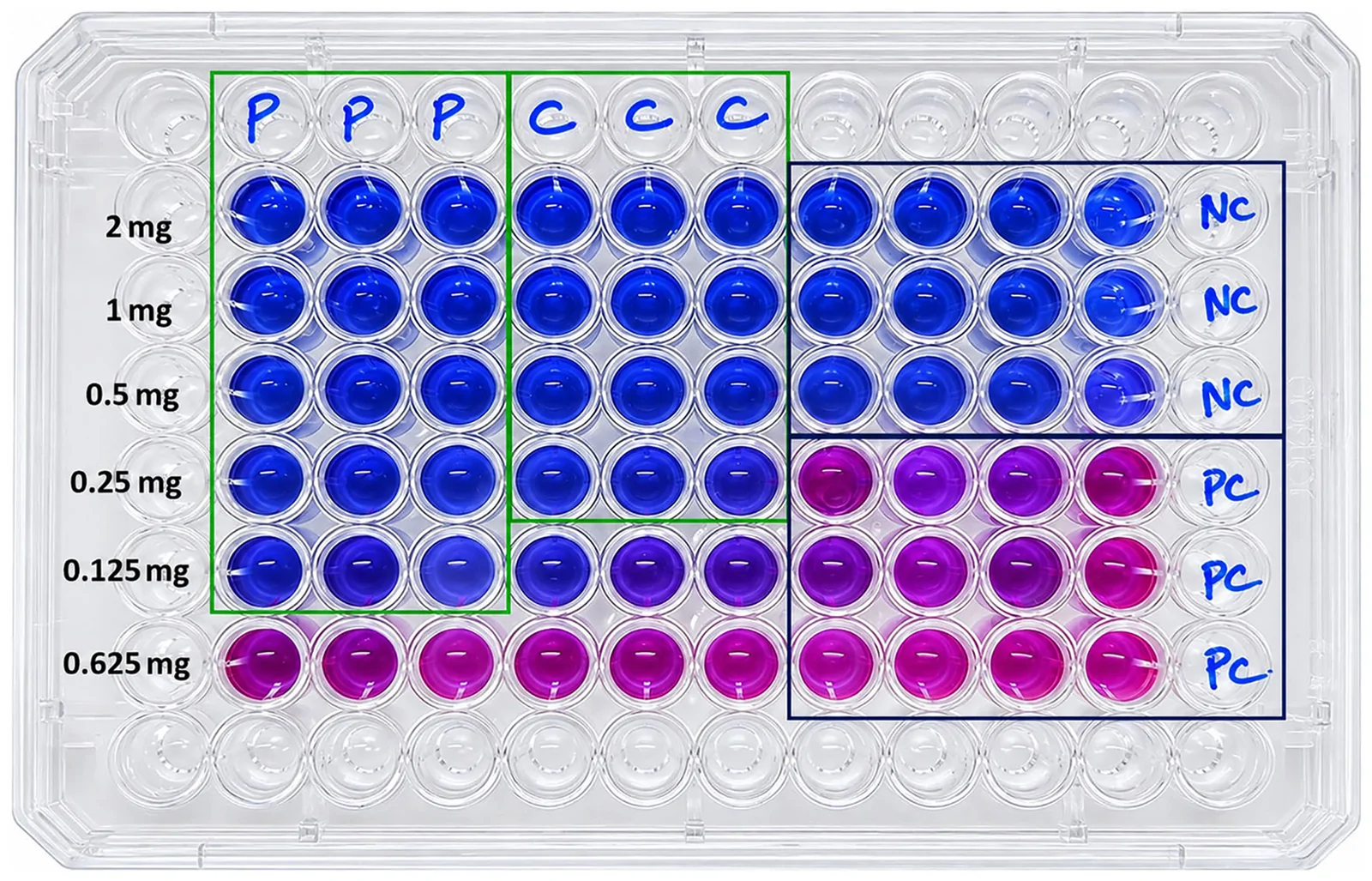
Determination of the MIC of *Piper longum* and *Cressa cretica* methanolic extracts against M. tuberculosis H37Rv by the resazurin microtitre assay (REMA). The assay was performed using extract concentrations ranging from 2 to 0.625 mg/ml. The MIC was defined as the lowest concentration showing complete inhibition of the resazurin colour change from blue to pink. The MIC was determined to be 0.125 mg/ml (125 µg/ml) for Piper longum and 0.25 mg/ml (250 µg/ml) for *Cressa cretica*. P, *Piper longum* (green box); C, *Cressa cretica* (green box); NC, negative control (black box); PC, positive control (black box). Data are shown for triplicate wells for each extract concentration. MIC, minimum inhibitory concentration.

**Figure 2:**
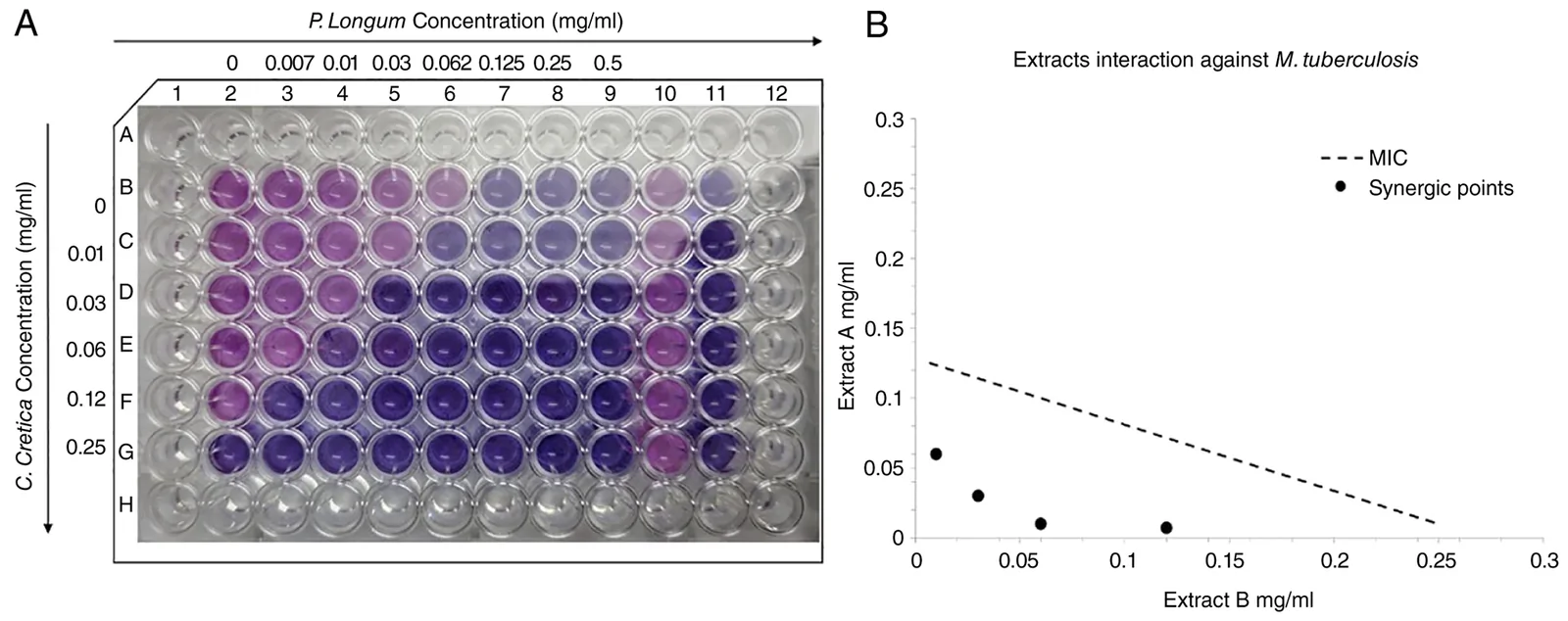
Combined effect of *P. longum* and *C. cretica* is shown by a checkerboard assay and isobolographic analysis. (A) The checkerboard assay is represented by columns representing different concentrations of *P. longum*, and the rows representing different concentrations of *C. cretica* to identify the most effective combinations for inhibiting *M. tuberculosis* growth. Purple denotes no growth, pink denotes growth. (B) Adaptation of isobolographic analysis where the various concentrations of *P. longum* and *C. cretica* are plotted on a graph represents their ability to inhibit the growth of *M. tuberculosis.* Combination of these extracts is more effective in inhibiting *M. tuberculosis* compared to independently. As plotted points fall below the expected line, it suggests a synergistic effect, indicating that the combination is more potent than individual use. Extract A represents *P. longum* and Extract B represents *C. cretica*, *P. longum*, *Piper longum*; *C. cretica*, *Cressa cretica*.

**Figure 3:**
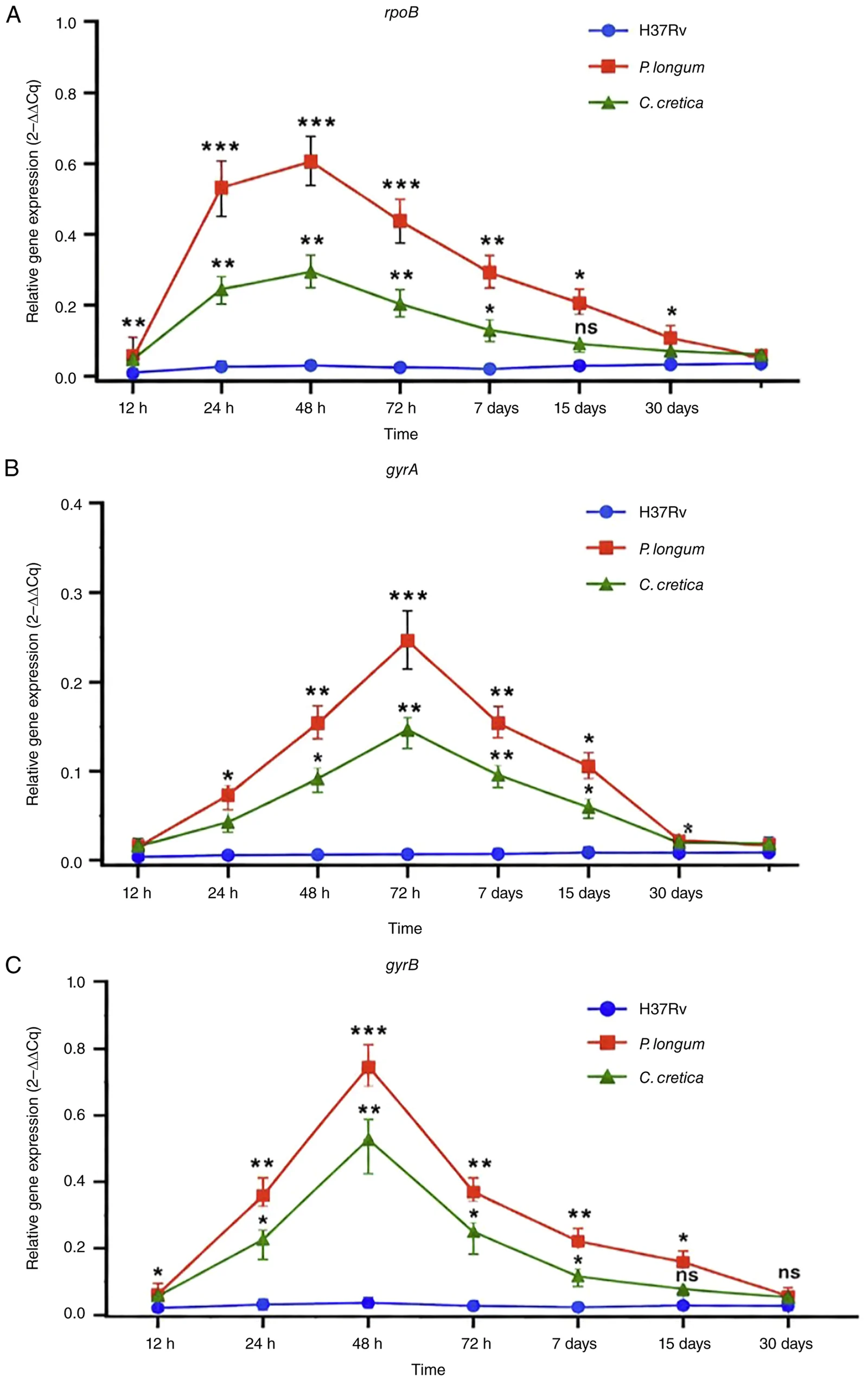
Time-dependent changes in relative expression of (A) *rpoB*, *(B) gyrA*, (C) and *gyrB* in *Mycobacterium tuberculosis* H37Rv following treatment with *Piper longum* and I extracts. Relative gene expression was determined using the comparative 2^-ΔΔCq^ method and normalized to the untreated H37Rv control. Data are presented as the mean ± SD from three independent experiments. Statistical analysis was performed using two-way ANOVA with treatment and time as the two factors, followed by Tukey's multiple-comparisons test. Comparisons were made between treatment groups at each time point and between different time points within each treatment group. ^*^P<0.05, ^**^P<0.005 and ^***^P<0.0005; ns, not significant. *P. longum*, *Piper longum*; *C. cretica*, *Cressa cretica*.

**Table I:** Extract preparation and dilutions.

			Extract dilution		
Extract/weight	Solvent/volume	Stock (mg/ml)	Stock (µl)	7H9 (µl)	Working solution
*P. longum* 100 mg	1% methanol 10 ml	10	300	1,200	2 mg/ml
*C. cretica* 100 mg	0.05% DMSO 10 ml	10	300	1,200	2 mg/ml

**Table II:** Primers used to amplify and sequence *rpoB*, *gyrB*, *gyrA*, 16SRNA and ITS.

Gene	Primer	Sequence (5'→3')	Amplicon size (bp)
*rpoB*	F	ACACCGCAGACGTTGATCA	369
	R	TCGTCGGCGGTCAGGTAC	
*gyrB*	F	GGCGCCGAAGTCGGCAAGT	157
	R	CCGGAAAAGTCCACGATGCTGCTA	
*gyrA*	F	CGTCGTTGTCACTATCACCG	207
	R	GATCAAATCGTGGGTGGAGC	
*16sRNA*	F	CCTTCGGGTTGTAAACCTCTTTC	132
	R	GGACAACGCTCGCACC	
*ITS*	F	TGCGGCTGGATCACCTCCTT	101
	R	CCGGCTCTCGCCCACTACAG	

F, forward primer; R, reverse primer; ITS, internal transcribed spacer.

**Table III:** Results of the phytochemical tests done of the three selected medicinal plant extracts.

Serial no.	Name of metabolites	*Piper longum*	*Cressa cretica*
1	Phenols	-	+
2	Terpenoids	+	-
3	Sterols	+	+
4	Tannins	-	+
5	Flavonoids	-	+
6	Alkaloids	+	+
8	Carbohydrates	+	+
9	Glycosides	-	+
10	Protein	+	+
11	Anthraquinone	-	-
12	Phytosteroids	-	-

The symbol ‘+’ indicates ‘present’, while the symbol ‘-’ indicates ‘absent’.

## Data Availability

The data generated in the present study may be requested from the corresponding author.
